# Human Influenza Virus Infection: A Focus on Key Host Determinants Linked to Clinical Disease Severity

**DOI:** 10.3390/v18030376

**Published:** 2026-03-18

**Authors:** Flora De Conto

**Affiliations:** Department of Medicine and Surgery, University of Parma, 43126 Parma, Italy; flora.deconto@unipr.it

**Keywords:** influenza virus, virus–host interaction, prevention measures, single-nucleotide polymorphism, severe influenza, host genetics, host cell determinants

## Abstract

Influenza viruses remain a major health threat, causing significant illness, death, and high healthcare costs worldwide, despite ongoing research and prevention efforts. A complex interaction between host and viral factors greatly influences the outcomes of influenza infection. Early research mainly focused on the influenza virus’s characteristics, but gaining an in-depth understanding of host factors involved in infection helps identify those that may influence disease severity. Notably, as the number of people with one or more comorbidities—that is, conditions that increase the risk of severe influenza—continues to rise, it becomes even more important to investigate the role of host factors. Recognizing host risk factors associated with severe outcomes, mainly caused by excessive inflammatory responses and disruption of epithelial barrier function, is crucial in identifying predictive markers and developing host-based prevention and treatment strategies, especially during pandemics. Moreover, early identification of host risk factors can help reduce severe outcomes and healthcare costs due to hospitalization. To achieve this, detailed analyses of the molecular signature of the host response to influenza virus infection are essential. This review highlights key host factors involved in severe influenza, allowing a better understanding of their roles, especially at the cellular level.

## 1. Introduction

The *Orthomyxoviridae* family comprises four different types of influenza viruses with negative-sense, single-stranded, segmented RNA genomes: influenza A and B viruses, responsible for classic influenza symptoms; influenza C virus, which has little clinical relevance; and influenza D virus, whose ability to infect humans is not yet fully understood. Influenza A viruses are further subtyped based on molecular differences in the two surface glycoproteins, hemagglutinin (HA, 19 subtypes), and neuraminidase (NA, 11 subtypes) [1,2].

Seasonal influenza is an acute respiratory infection with high transmissibility and global distribution. The infection mainly spreads through tiny droplets when infected people cough, sneeze, or converse. Influenza is characterized by a variety of symptoms (fever, cough, sore throat, runny or stuffy nose, muscle or body aches, headaches, and fatigue), involving the upper and lower respiratory tracts [3].

Nearly 1 billion cases of seasonal influenza occur annually, including 3–5 million cases of severe illness. Influenza viruses also pose a pandemic threat, alongside periodic epidemics. Noteworthily, influenza results in 290,000 to 650,000 respiratory deaths annually [4].

The most common complication of influenza is a bacterial infection, which affects the respiratory system and can, in turn, lead to bronchitis and develop into pneumonia. It can also cause ear infections (otitis) and sinusitis, especially in children. In particular, influenza-related pneumonia is a serious complication responsible for over 50% of hospitalizations. The disease may degenerate into severe respiratory infections, leading to respiratory failure and acute respiratory distress syndrome, which are generally correlated with an exaggerated inflammatory response. Additionally, influenza can cause extra-pulmonary complications, involving the cardiovascular system (such as myocarditis) and the nervous system, and can also worsen preexisting health conditions. Moreover, hospitalization for severe influenza cases incurs extremely high economic costs for the healthcare system [5,6].

Influenza A viruses (IAVs) can also infect various animal species and are seen as representing a significant zoonotic risk. As a result, there is increasing concern over the potential transmission of highly pathogenic avian strains, such as subtypes H5, H7, and H9, to humans [7]. In this regard, previous data assessed the involvement of host cell cytoskeleton components during infection of mammalian cells by avian influenza A virus strains [8].

The epidemiology of influenza primarily depends on the virus’s tendency to mutate, which involves antigenic changes developing in the two surface glycoproteins, HA and NA. These changes allow the viruses to evade the immune response from previous infections, enabling them to infect a large part of the susceptible population and spread quickly. However, it is important to remember that, on its own, the appearance of a strain with completely new surface proteins does not necessarily cause a pandemic; the new influenza virus must also be able to transmit efficiently from person to person.

The most crucial step in preventing influenza is to enhance biosafety protocols and receive an annual influenza vaccination [3,9]. In fact, the seasonal influenza vaccine has been shown to greatly lower hospitalization and death rates, particularly among vulnerable populations [9,10,11,12]. Additionally, two types of antiviral drugs are currently used to treat influenza: neuraminidase inhibitors (oseltamivir and zanamivir) and other drugs which inhibit the viral M2 protein (amantadine and rimantadine). However, such therapies are likely to be effective in limited groups of the population and during specific stages of an influenza infection.

The emergence of a new or unexpected influenza virus strain commonly presents a major challenge since no vaccine is readily available in the early phases of a pandemic. Furthermore, increased drug resistance and the zoonotic potential of influenza viruses underscore an urgent need to develop innovative broad-spectrum vaccines [13,14,15].

To date, most attention in research plans has focused on assessing virulence determinants of influenza viruses, while there is a lack of in-depth studies, particularly at the cellular level, looking at risk host factors that can worsen disease outcomes.

This review focuses on the role of individual key features and cellular determinants that promote the development of severe outcomes during influenza virus infection. Predicting which individuals are at higher risk of developing severe influenza to institute personalized prevention plans and medical interventions requires thorough analyses of the molecular signatures of the host response during viral infection.

## 2. Individual Subject Factors That May Predict a Severe Outcome of an Influenza Virus Infection

The Centers for Disease Control and Prevention (CDC) has identified several population groups at higher risk for serious influenza complications, including adults aged 65 and older, children under 2 years, people with chronic illnesses, and those with weakened immune system responses [9]. Additionally, it has been shown that certain racial and ethnic groups have experienced major severe outcomes from influenza [16,17]. Vaccination and antiviral treatments are strongly recommended for individuals at high risk of influenza-related diseases [18].

Obesity is a major risk factor among comorbidities that affect the host’s likelihood of developing severe influenza, causing high hospitalization rates [19,20]. Obesity is often correlated with an altered inflammatory profile, characterized by a chronic state of meta-inflammation that affects the entire system, impacting immunity and slowing down antiviral responses to influenza virus infection [21,22]. Worryingly, obesity is an increasing trend, especially among children.

Most experimental influenza studies on obesity have been conducted in mouse models. The results in obese mice have highlighted a poor innate immune response to influenza infection with increased expression of proinflammatory cytokines and chemokines, causing an increased flux of inflammatory cells [23,24]. Obese mice show enhanced expression of cytokine signaling suppressor mRNA in the lung, resulting in lower levels of type I interferon (IFN) [25]. Additionally, obesity impacts the adaptive immune system, with reduced levels of pulmonary CD4+ T cells having been observed [26]. Interestingly, it has recently been suggested that obesity may also influence IAV genetic diversity, creating a unique selective environment that affects viral evolution and spread [27].

It must also be considered that obesity is often linked to other chronic disorders, such as diabetes, as well as cardiovascular, renal, hematological, and pulmonary diseases, which are also independently involved in the development of severe influenza, as shown in multicenter surveillance studies, involving both hospitalized subjects and outpatients [28,29,30,31].

A host’s chronic or temporary immunocompromised state is considered an important risk factor, making individuals more prone to influenza complications and hospitalizations [32,33,34], as shown in studies conducted in large cohorts of hospitalized patients, and considering that in these subjects, influenza vaccination is less effective [35,36].

A prolonged bedridden condition has also been strongly linked to mortality among patients hospitalized with severe acute respiratory infection, including influenza [37]. These patients have exhibited high rates of hypoalbuminemia and undernutrition, linked to impaired or absent swallowing and cough reflexes [38].

Advanced age is another major risk factor for severe influenza, as both innate and adaptive immune responses are negatively impacted, as shown in studies conducted in large cohorts of subjects aimed at evaluating their cellular immune profile [39,40]. Importantly, immunosenescence involves significant changes in lung macrophages, leading to increased pulmonary fibrosis and negatively affecting phagocytosis [41,42].

It has also been accepted that very young children and children with underlying medical conditions, such as asthma, are at higher risk of severe influenza complications, re-hospitalization, and death, as shown in meta-analysis studies as well as in studies involving cohorts of re-hospitalized children [43,44,45]. This is primarily because of the immune system’s functional immaturity. Importantly, research has shown that single-gene mutations impacting the interferon pathway led to severe influenza infections in children [46].

It has been argued that immunity varies between the sexes. Accordingly, females exhibit stronger immune responses to influenza vaccination [47]. However, pregnancy is considered a risk factor for developing severe influenza, with a higher chance of hospitalization after infection compared to nonpregnant women, as emerges from surveillance studies on pregnant women, based on data reported to the CDC [48,49]. In pregnancy, increased expression of pro-inflammatory cytokines, elevated levels of suppressive cytokines, and depletion of type I and III IFNs contribute to severe immunopathology [50,51,52]. It has also been shown that CD8+ and CD4+ T cells decrease in number and efficiency during IAV H1N1 infection [53].

**Figure 1:**
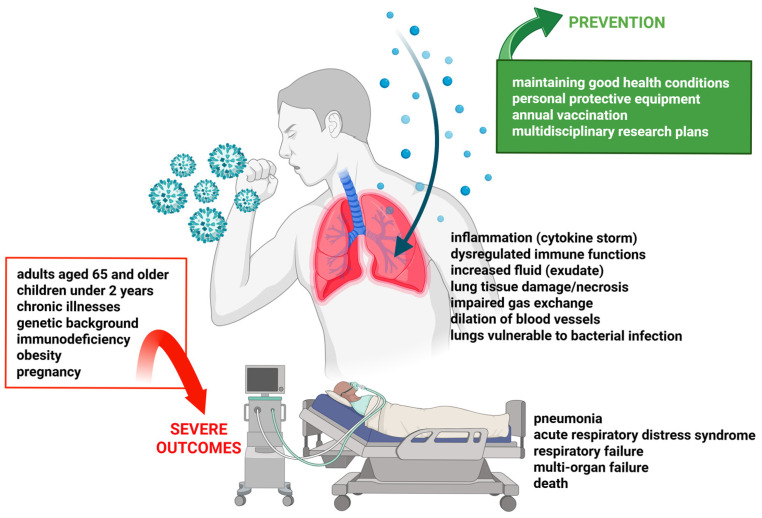
Individual risk factors that promote the severe course of influenza infection. The main individual risk factors that negatively impact the course of influenza virus infection, causing the most severe effects, are shown. These factors can act individually or synergistically, affecting host defenses. Permanent or temporary immunosuppression is a primary risk factor for the onset of severe infections. Similarly, extremes of life span correlate with impaired immune responses. Obesity causes tissue inflammation that depresses antiviral responses; this condition often acts as a synergistic factor when associated with chronic diseases, which can also cause severe infections individually. Pregnancy causes a significant dysregulation of the immune response, favoring the onset of severe infections. Created in BioRender. De Conto, F. (2026) https://BioRender.com/wum7ry7 (accessed on 17 February 2026).

Figure 1 shows the most relevant individual risk factors that promote severe influenza along with possible repercussions on human health.

Given the observed increase in the above-mentioned comorbidities, there is a growing risk of developing high rates of severe influenza cases leading to hospitalizations and deaths. Therefore, prevention measures and healthcare must be intensified for vulnerable subjects, and intervention plans based on personalized medicine [54].

## 3. Host Genetic Factors Impacting Influenza Severity

The severity of influenza varies among individuals. Comorbidities can only account for a small part of severe influenza cases and do not explain those seen in healthy individuals [55]. In this regard, a specific host genetic background may be influencing susceptibility and the outcomes of influenza virus infection through innate and adaptive immune responses [56].

Genetic polymorphisms differ among individuals, typically involving a single nucleotide substitution (i.e., single-nucleotide polymorphism, SNP). In this review, the significance of major SNPs in relation to the pathogenesis of influenza is discussed.

Among the most studied genetic factors, variants in genes regulating the IFN responses (see Table 1) prove crucial for ensuring early antiviral host defense.

In this context, rare genetic variants in genes that modulate the IFN-1 cytokine family responses have been examined in cases of severe influenza [46,82,83]. Specifically, common variants in the *DDX58* gene, which encode immune signaling components of the retinoic acid-inducible gene I receptor, may represent a pediatric risk factor for severe influenza, as shown in studies involving both hospitalized and non-hospitalized subjects [84,85]. Moreover, a significant association has been found between the minor interferon-induced transmembrane protein-3 (IFITM3) genetic variant SNP rs12252-C and susceptibility to severe influenza [86,87]. The *IFITM3* gene is crucial in protecting the host against IAV. Moreover, *IFITM3* limits the replication of various pathogenic viruses that enter cells through endocytosis by altering membrane dynamics to prevent fusion between viral and host membranes [88,89]. Specifically, in three cohorts of subjects characterized by different levels of influenza illness severity, it has been found that carriers of the risk allele rs34481144 in the *IFITM3* gene had fewer CD8+ T cells in their airways during influenza infection, suggesting that a critical role for *IFITM3* may be that of supporting immune cell persistence at mucosal sites [90]. The SNP rs12252-C, common in Asian populations, creates a splice acceptor site, leading to truncated IFITM3 and decreasing its ability to inhibit influenza infection [91,92]. Furthermore, another SNP in the *IFITM3* gene, rs34481144-A, common among European populations, reduces mRNA and protein levels [88,90]. Importantly, studies carried out in mouse models evidenced that IFITM3 deficiency lowers the infectious dose needed to establish a productive infection with zoonotic influenza strains, and that passaging through IFITM3-deficient hosts accelerates interspecies influenza transmission, emphasizing the crucial role of IFITM3 in pandemic prevention strategies [88,93].

Most patients infected with the IAV H1N1 2009 pandemic virus showed increased systemic levels of pro-inflammatory cytokines. It has been shown that genetic variants of interleukin-1A (IL-1A) and IL-1B may significantly influence susceptibility to IAV H1N1 virus infection [94]. Moreover, gene polymorphisms on chromosomes 1 and 17 might affect the risk of developing severe pneumonia. Specifically, two SNPs are located within genes *FCGR2A* and *C1QBP*, which are chiefly involved in regulating immune complexes and complement activation, indicating heightened activation of the host immune response, as evidenced in subjects with severe pneumonia [95]. In addition, SNPs in the tumor necrosis factor-alpha (*TNF-α*) gene have also been linked to severe influenza [96].

Several immunogenetic factors have been correlated to an increased risk of developing severe IAV H1N1 pandemic symptoms, including CCR5, KIR, IFITM3, and IGHG2 [97,98]. Li et al. [99], using host-based whole-genome sequencing in blood samples from subjects with influenza to explore genetic risk loci associated with the severity of IAV H1N1 2009 infection, identified SNPs located in the polypyrimidine tract-binding protein 3 (*PTBP3*) gene, belonging to the family of RNA-binding proteins and acting on cell differentiation. These same authors also investigated other genes that could increase the risk of severe influenza, such as *FTSJ3*, *CPVL*, *BST2*, *NOD2*, and *MAVS*. Moreover, complement-related SNPs, the rare TT genotype of *CD55*, and the rare AA genotype of *C1QBP* were significantly associated with an increased risk of death upon IAV infection [100].

Interestingly, a recent in-depth genome-wide association multicenter study bearing on influenza virus infection, and analyzing a large cohort of subjects, showed no significant links between genetic risk factors for influenza and COVID-19 diseases, respectively [101]. Specifically, this study identified two risk variants for influenza in or near the *B3GALT5* and *ST6GAL1* genes. Accordingly, inhibiting *ST6GAL1* experimentally, which depletes the enzyme β-galactoside α-2,6-sialyltransferase 1, reduces influenza virus replication.

The genetic features of human populations are key in risk assessment and predicting susceptibility to severe influenza, although their impact may vary depending on specific host-related factors, such as age and ethnic variations [86,102]. Consequently, more detailed research involving large populations of different ethnicities and age groups is needed.

## 4. Host Cell Determinants Modulating the Pathogenesis of Influenza Virus Infection

Numerous host cell factors/functions are vital for stimulating the virulence of the influenza virus and causing severe disease. Interestingly, recent studies have highlighted the ability of the influenza virus to subvert host cell metabolism to promote its replication, causing a reprogramming of cellular metabolism with negative repercussions on the response to infection [103,104].

Among the host cell determinants involved, some of them act by enhancing influenza virus replication and causing negative repercussions on host defense functions. In this regard, Bcl-2-interacting killer (BIK) protein deficiency hampers IAV replication, while BIK overexpression increases viral load, inflammation, and mortality in influenza virus-infected mice [105]. Specifically, IAV can suppress β5, a proteasome subunit, through its nucleoprotein, leading to increased BIK levels and enhanced replication. Of note, the variation rs738276 in the *BIK* gene has been associated with influenza severity in humans [105,106]. Overall, these findings identify the pro-apoptotic BIK protein as a key host factor affecting influenza severity.

Hypoxia-inducible factor-1α (HIF-1α) is another cell determinant supporting IAV H1N1 virus replication and leading to the activation of a cytokine storm in alveolar epithelial cells [107]. Deficiency of HIF-1α reduces pulmonary injury, viral replication, and cytokine storms in vivo, while its upregulation, induced by H1N1 infection, enhances viral replication by reprogramming the cellular metabolism toward glycolysis. Although these metabolic changes are aimed at supplying energy for H1N1 virus replication, a parallel IFN-α/β inhibition has been observed.

To promote their replication, IAVs recruit specific cell components of the nuclear import pathway. Among these, the importin-α7 protein favors the pulmonary tropism of H1N1 IAV by enhancing cytokine and chemokine levels, mononuclear infiltration, and alveolar destruction, and causing severe pneumonia and death in mice [108]. Accordingly, importin-α7-deficient mice showed influenza infection restricted to the bronchial epithelium and improved survival rates.

Moreover, the tetraspanin Cluster of Differentiation 151 (CD151) protein, which is crucial for cell adhesion, migration, and maintaining tissue integrity, and is particularly expressed in the lungs, is involved in nuclear influenza virus export signaling. CD151 acts by binding to newly synthesized viral proteins and the host’s nuclear export protein CRM1 [109,110]. CD151-deficient infected mice exhibited a significant reduction in influenza virus replication and improved survival rates, consequent to a pronounced host antiviral response and inflammasome activation [110]. Therefore, treatments targeting CD151 may be affecting relevant host mechanisms involved in IAV signaling, in the meantime, bypassing the frequent antigenic changes observed in influenza virus strains, which hamper the preparation of broad-spectrum vaccines.

Influenza is primarily correlated with inappropriate activation of the innate immune responses, causing detrimental lung inflammation. Accordingly, influenza virus pathogenesis not only depends on the viral replicative efficiency and occurrence of the cytopathic effect, but also on the exacerbated host inflammatory response, due to the hyperinduction of interferons and proinflammatory cytokines. It is worth mentioning that the pathways promoting excessive innate immune system activation and the strictly involved cell factors are insufficiently explored.

Among the cell factors/functions leading to dysregulation of immune system responses upon influenza virus infection, with negative repercussions on the functionality of the respiratory system, interleukin 6 (IL-6) is considered a primary determinant of inflammation regulation. Excessive IL-6 production and upregulated expression of the suppressor of cytokine signaling-3 (SOCS3) during influenza virus infection contribute to immune response dysregulation and the occurrence of a cytokine storm; overall, these events cause tissue damage and lead to severe outcomes [111,112,113]. In addition, IL-36γ shows higher expression levels in patients with acute respiratory distress syndrome caused by the influenza virus. Specifically, IL-36γ enhances type I and III IFNs, indicating its primary regulatory activity in the IFN signaling pathway [114]. Moreover, it has been assessed that the translocase of the outer mitochondrial membrane (TOMM)—a multimeric protein complex responsible for recognizing and importing mitochondrial preproteins from the cytosol—serves as a key signaling modulator of antiviral innate immunity, facilitating the transmission of antiviral signals. In severely ill influenza patients, there is an observed upregulation of TOMM34 transcription in circulating monocytes, lung epithelium, and innate immune cells induced by IAV [115]. This perspective also includes the fact that IAVs can reduce expression levels of the eukaryotic translation initiation factor 48 (eIF48) through lysosomal degradation mediated by the viral protein NS1, as evidenced in cell cultures and mice [116]. EIF48 transgenic mice were more susceptible to IAV infection, exhibiting shorter survival time and severe organ damage. In turn, eIF48 has been observed to have a regulatory effect on IFITM3, which is responsible for antiviral activity, as previously reported. Therefore, IAVs have seemingly acquired the ability to overcome innate immunity by downregulating the expression of the cellular factor eIF48.

One key determinant in the activation of a cytokine storm has been observed to be the interferon-induced protein 35 (IFP35), which is released into lung epithelial and alveolar cells upon influenza virus infection. Experimental studies have demonstrated that its inhibition with neutralizing antibodies reduces lung damage and post-infection mortality [117]. Moreover, it has been appraised that nuclear matrix protein 4 promotes monocyte- and neutrophil-attracting chemokine expression upon IAV infection, causing exaggerated inflammation and lung damage [118].

The tripartite motif-containing 28 (TRIM28/KAP1/TIF1β) is a critical regulator of IFN-β, IFN-γ, and cytokine expression during infection with highly pathogenic IAVs in human lung cells [119]. Specifically, these viruses trigger TRIM28 phosphorylation at serine 473, enhancing IFN-β and proinflammatory cytokine levels during infection. Therefore, these observations could well serve as a starting point for the development of new immunomodulatory strategies by targeting TRIM28 post-translational modifications to control the expression of type I interferons, as well as that of proinflammatory cytokines.

Integrins are surface receptors that sense extracellular changes to activate intracellular signaling cascades. In obese-mouse models, the lungs show increased expression of epithelial cell β6 integrin, a host factor linked to influenza disease severity [120]. Knockout of the β6 integrin in the lung during influenza infection restores homeostatic levels and increases IFN-1 signaling, favoring host survival. Therefore, the loss of β6 leads to a heightened antiviral state, which restricts viral spread and results in less acute lung injury. Recent findings have assessed that activation of the epithelial β6 integrin during IAV infection is linked to innate immune impairments [121]. Specifically, β6 modulates the Toll-like receptor 7 (TLR7) through the regulation of the intracellular trafficking, leading to its reduction in endosomal compartments and the associated TLR7 signaling.

Tumor progression locus 2 (TPL2) is a serine-threonine kinase that enhances inflammation. TPL2-deficient mice succumb to infection with a H3N2 low-pathogenicity strain of influenza virus, while no virus has been observed in their lungs on the day of peak morbidity, suggesting an overactive antiviral immune response [122]. Accordingly, elevated cytokine and chemokine levels appear to have been accompanied by increased infiltration of the lungs and correlated with increased IFN-inducible monocyte chemoattractant protein-1 and nitric oxide synthase expression, which has been associated with severe influenza. These observations suggest that TPL2 tempers inflammation during influenza infection by constraining the production of interferons and chemokines. Importantly, other findings obtained in mice have demonstrated the protective role of TPL2 in influenza virus pathogenesis and revealed that TPL2-deficiency is sufficient to convert a low-pathogenicity IAV infection into a severe disease [123]. These findings warrant in-depth studies to set up molecular strategies able to modulate TPL2 function during viral infection [124].

The acidic leucine-rich nuclear phosphoprotein 32B (ANP32B) is an essential component of the influenza virus replication machinery, which promotes IAV pathogenesis in mice and shows immune-modulatory functions [125]. Replication of avian IAVs in mammalian cells is hindered by species-specific variation in ANP32, which is essential for viral RNA genome replication. Notably, the occurrence of adaptive mutations enables the IAV RNA polymerase to surmount this barrier to favor mammalian adaptation of avian IAVs [126].

Among cell functions involved in infectious processes, mitophagy serves as a host defense mechanism. Recent data have shown its stronger activation after IAV H1N1 infection and subsequent superinfection with *Staphylococcus aureus* in A549 lung epithelial cells, involving the PINK1/Parkin signaling pathway [127]. The activation of mitophagy correlates with increased bacterial and viral loads, worsening inflammation, and promoting the development of severe pneumonia. Additionally, it has been found that greater severity in IAV infection and a reduced innate immune response are also associated with extensive mitophagy through a PINK1 pathway-independent mechanism in senescent human cells. In this context, the apolipoprotein D was identified as being significantly elevated in the lungs and sera of aged subjects, in which it suppresses the type I IFN response and increases infection severity [128].

Another cellular factor that affects a host’s antiviral response against IAV infection is the Programmed Cell Death Protein 1/Programmed Cell Death Ligand 1 (PD-1/PD-L1) pathway. Severe influenza induces PD-1/PD-L1 expression, and its levels correlate to the extent of pathological damage in the lungs of mice [129]. Severe infection has been associated with increased PD-1 expression on influenza virus-specific CD8+ T cells and with a dysregulated CD8+ T cell response, likely caused by the more highly inflamed airway microenvironment. In fact, it has been assessed that during a severe influenza virus infection, CD8+ T cells are less abundant and functional than in a milder infection. This observation is correlated with a lower rate of virus clearance in severe infection and is partially regulated through the expression of PD-1. Notably, treatments neutralizing PD-1 improve T cell functionality and increase virus clearance [130].

Forbester et al. [131] showed that interferon regulator factor 5 (IRF5) deficiency in human stem cell-derived dendritic cells or macrophages reduced cytokine production. IRF5 is a transcription factor that acts as a regulator of myeloid cell inflammatory cytokine production, driving immune-mediated influenza virus pathogenesis. Additionally, another study revealed that, in patients with community-acquired pneumonia caused by influenza virus infection, the levels of IRF5 and IFN-α increased significantly in the early phase of pneumonia [132]. Overall, these data suggest that genetic variation in the *IRF5* gene may influence host susceptibility to viral diseases.

Moreover, it has been assessed that imbalanced cell glycosylation can alter viral glycome, resulting in weakened immune responses. Individuals with conditions such as diabetes, obesity, and immune disorders often exhibit changes in cellular glycosylation and tend to experience more severe influenza. Abnormal glycosylation may increase the risk of severe influenza since glycome-modified influenza viruses can evade immune responses [101]. Moreover, it has been shown that glycan epitope high mannose during a host response to influenza virus infection can be considered a marker of the severity of the disease [133].

Finally, other studies have demonstrated the involvement of certain cellular factors that promote the invasiveness of influenza viruses, inducing lung injury. In this regard, influenza virus infection in vitro and in mice upregulates the expression of cathepsin S (CTSS), a lysosomal cysteine protease, leading to lysosomal membrane permeabilization, an increase in apoptosis, and loss of epithelial barrier integrity [134]. Accordingly, it has been shown that inhibition of CTSS alleviates influenza severity in infected mice. In addition, influenza virus infection in alveolar macrophages leads to impaired mitochondrial transcription factor A, diminishing mitochondrial efficiency and causing accumulation of surfactant and cellular debris, which increases host susceptibility to severe influenza [135].

**Figure 2:**
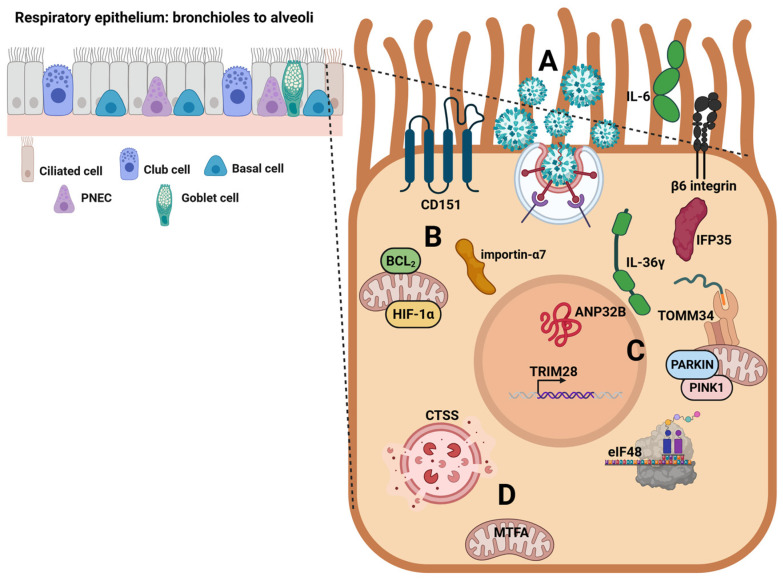
Influenza virus hijacks key host cell determinants in respiratory epithelial cells to promote severe disease. **A.** Influenza virus infection in respiratory ciliated epithelial cells stimulates specific host cell factors, promoting severe pathogenesis. **B.** Host cell factors promoting influenza virus replication and affecting host defenses: Bcl-2-interacting killer (BIK) protein increases viral load and inflammation; hypoxia-inducible factor-1α (HIF-1α) protein supports virus replication and leads to the activation of a cytokine storm; importin-α7 protein favors the pulmonary tropism; tetraspanin Cluster of Differentiation 151 (CD151) protein regulates nuclear virus export signaling. **C.** Host cell factors leading to dysregulation of immune system responses affecting the functionality of the respiratory system: interleukin 6 (IL-6) contributes to immune response dysregulation and occurrence of a cytokine storm; interleukin 36γ (IL-36γ) enhances type I and III IFNs levels; translocase of the outer mitochondrial membrane 34 (TOMM34) modulates innate immunity; eukaryotic translation initiation factor 48 (eIF48) counteracts innate immunity; interferon-induced protein 35 (IFP35) activates the cytokine storm; tripartite motif-containing 28 (TRIM28) regulates IFN-β, IFN-γ, and cytokine expression; β6 integrin impairs innate immune defenses; acidic leucine-rich nuclear phosphoprotein 32B (ANP32B) favors viral replication; PARKIN/PINK activation correlates with increased viral loads and inflammation. **D.** Host cell factors promoting the invasiveness of influenza viruses and lung injury: cathepsin S (CTSS), a lysosomal protease, leads to the loss of epithelial barrier integrity; mitochondrial transcription factor A (MTFA) diminishes the mitochondrial efficiency, causing accumulation of surfactant and cellular debris. PNEC: pulmonary neuroendocrine cells. Created in BioRender. De Conto, F. (2026) https://BioRender.com/f7exj3h (accessed on 17 February 2026).

Figure 2 shows the main host cell determinants hijacked by the influenza virus in respiratory epithelial cells in the case of severe disease.

Overall, the reported data suggest that the influenza virus can co-opt several cellular factors that promote viral replication and subvert host defense mechanisms, heralding a severe course of the viral infection. These observations, which highlight the complexity of the virus–host interaction, warrant further investigation with large-scale translational studies to assess whether these host cell determinants could constitute promising targets for the development of new broad-spectrum and personalized medical interventions.

## 5. The Current Influenza Epidemic: Prevention Measures and Treatments

Regarding the current influenza season, it must be noted that late outbreaks of influenza A (H3N2) virus subclade K have been observed in Australia and New Zealand, suggesting that it could also eventually impact the Northern Hemisphere. Indeed, subclade K viruses have been detected in over 34 countries and seem to have spread worldwide, except for South America [136]. In Europe, the influenza season began in October 2025, four weeks earlier than the previous two seasons, driven by the emergence of the influenza A (H3N2) virus subclade K [137,138]. As of week 49, 2025, there has been significant circulation of several respiratory viruses, with IAV dominating across all European countries, and mainly affecting children aged 5–14 years. Notably, there has been an increase in hospitalizations, chiefly among adults aged 65 and older [137]. The CDC indicates that there have been at least 20 million illnesses, 270,000 hospitalizations, and 11,000 deaths up to 24 January 2026 [139]. Globally, influenza activity remains high, with positivity just under 20% in week 2 of 2026 [140].

Vaccination against the influenza virus is considered the most effective method for preventing severe outcomes of the disease [141,142]. It also has to be considered that influenza vaccination provides substantial benefit in reducing infections in both the vaccinated and unvaccinated portions of the population. Krauland et al. [143] have shown that even when both vaccine effectiveness and vaccine uptake were low, influenza vaccination showed marked reductions in disease burden.

The CDC’s issues on the 2025/26 influenza season confirm the need to periodically update recommendations regarding the administration of an influenza vaccine to strengthen disease prevention and control [144]. Accordingly, Italian guidelines are revised annually to ensure the administration of the most appropriate vaccine, based on age and health status. Importantly, the concept of “precise vaccination” is becoming more widespread to enhance protection in high-risk individuals [145]. In recent years, increasing attention has been given to confer the most suitable vaccine to each population group. The most relevant result being the near elimination of suboptimal vaccine administration in the elderly population [146].

Although vaccination lowers the risk of severe disease, hospitalization, intensive care, and mortality, in recent years, public opinion has increasingly viewed vaccinations as ineffective and unsafe, due to a complex interplay of cultural, social, and political influences [147]. Accordingly, vaccine hesitancy was among the World Health Organization’s top 10 global health threats in 2019 [148].

Regarding influenza vaccine effectiveness, it has been assessed that this is lower than that of other routine vaccines, varying from season to season between the Northern and Southern hemispheres, and can be modest in some seasons [149]. Evaluating vaccine effectiveness is critical in understanding the risks and benefits of timely vaccination programs. With this in mind, a multicenter study carried out in Europe in the 2023/24 season evidenced protection that was high against influenza B/Victoria, but lower against influenza A (H1N1) pandemic 2009 and influenza A (H3N2) viruses [150].

As regards the current influenza season, there have been some concerns over the effectiveness of the vaccines, due to a mismatch between the vaccine and the new subclade K, since its mutations allow the influenza virus to evade some of the influenza vaccine’s protection. Preliminary data indicate that the seasonal vaccines available in the European Union give protection against influenza A (H3N2) infection, with 52–57% effectiveness [137]. These results are consistent with other findings related to Northern Europe [151,152], supporting global efforts to offer seasonal influenza vaccination to key populations as early as possible. However, 41.3% vaccination effectiveness has been observed in China [153], indicating moderate protection during this subclade K-dominated season.

On the other hand, it is important to note that seasonal influenza vaccine uptake rates in high-risk groups are low. In this regard, during the 2024–2025 influenza season, the United States experienced the highest influenza case numbers since 2009, but with low vaccination uptake. Accordingly, the American Society of Behavioral Medicine recommends maintaining research funding, investing in vaccination promotion, and employing public health data sources to lessen the impact of influenza [154]. Specifically, a study carried out in the United States during the 2024–2025 influenza season showed that among hospitalized patients, only 32.4% of them had been vaccinated against influenza, while 84.8% had received antiviral treatment. Moreover, most patients hospitalized (89.1%) had one or more co-morbidities; of these, 16.8% were admitted to an intensive care unit, 6.1% received invasive mechanical ventilation, and 3.0% died in the hospital [155]. Meanwhile, Lin et al. [156] showed that influenza vaccination uptake decreased during the COVID-19 pandemic among children and healthcare personnel. Furthermore, Ma et al. [157] showed that influenza vaccination rates among older adults in mainland China are low, especially among those subjects lacking access to free policies and those with chronic diseases. Overall, these observations suggest that further studies are necessary to identify reasons for low compliance with influenza vaccine uptake. In the meantime, tailored strategies to improve seasonal vaccination in high-risk individuals must be set up and implemented to prevent illness, hospitalization, and death.

Treating severe influenza requires a multidisciplinary approach which combines antiviral and immunomodulatory strategies. One of the main obstacles in any antiviral setup is the genomic variability of influenza viruses, which constantly sees drug-resistance and immune evasion phenomena. Currently, developing effective therapies for severe influenza remains extremely challenging. In fact, no immunomodulatory agents have been definitively shown to provide benefits in severe cases. The World Health Organization provisionally advises against using systemic corticosteroids, macrolides, plasma therapy, mechanistic target of rapamycin inhibitors, and nonsteroidal anti-inflammatory drugs for critically ill patients [158]. Indeed, high-dose systemic corticosteroids may increase the risk of death and complications in severe influenza cases, while passive immunotherapy with convalescent plasma or intravenous immunoglobulin from healthy donors has not proven effective [159].

Recent issues have highlighted the role of some innovative anti-influenza therapeutic approaches, such as combination therapies and targeted protein degradation [160]. However, large-scale clinical trials are needed to confirm the efficacy of these ostensibly promising treatments. 

## 6. Discussion

Influenza represents a significant global public health challenge, disproportionately affecting more vulnerable populations.

Despite global prevention efforts and available treatments, considering the rapid evolution of influenza viruses, which limits the efficacy of vaccines and drugs, it is essential to search for broad-spectrum biomarkers to identify high-risk individuals and formulate personalized medicine interventions to reduce the incidence of severe infections. Accordingly, in this review, the roles of key host determinants linked to severe influenza, summarized in Table 2, have been examined.

Among the host factors shown in Table 2, those closely related to the individual subject have been extensively investigated in several multicenter surveillance studies, involving large cohorts of subjects. Therefore, their role in promoting the onset of severe influenza has been widely confirmed. Likely, the combined effect of two or more individual subject factors in causing severe influenza will tend to be increasingly common. This trend results from the increase in immunosuppressive treatments and the aging population, due to declining birth rates in various parts of the world, which leads to a higher rate of multimorbid patients [161,162,163,164]. To counteract the effects of individual host factors in favoring severe influenza, in addition to promoting a healthy lifestyle, it is necessary to strengthen influenza vaccination campaigns.

Regarding host genetic factors, the reported data were obtained either from studies conducted in vitro/mouse models or involving cohorts of subjects with severe influenza. Notably, these latter studies showed the relationship between SNPs detected in *DDX58*, *IFITM3*, *interleukin-1A* and *-1B*, *FCGR2A*, *C1QBP*, *PTBP3*, *B3GALT5*, and *ST6GAL1* genes and human severe influenza [84,85,90,94,95,99,101]. These observations highlight the need to develop laboratory diagnostic tools for detecting predictive genetic biomarkers. Moreover, SNPs carrier patients must be vaccinated to prevent severe influenza, and, in case of infection, personalized treatments should be envisaged.

The impact of the influenza virus on a host cell is multifaceted, taking advantage of a wide range of host cell factors and functions that facilitate the infectious process. On the other hand, the host cell activates numerous defense mechanisms to counteract the changes caused by the influenza virus, triggering a complex virus–host interplay that influences the infection’s outcome. Therefore, clarifying the mechanisms of interaction between the influenza virus and host cells at the molecular level may highlight how certain cellular factors contribute to the onset of severe forms of infection. Although knowledge on the influenza virus’s ability to subvert numerous cellular functions is increasing, insufficient attention has been paid so far to the role of cellular factors that selectively influence the severity of viral infection.

Intending to improve knowledge on proactively preventing and managing severe influenza infections, this review focuses on the role carried out by various host cell factors and the complex biological mechanisms regulating them (see Figure 2 and Table 2). Some of these factors affect the initial phases of influenza virus infection, promoting virus replication and modulating host defenses by stimulating excessive inflammatory responses. Other cellular factors act in a subsequent stage, either leading to immune system dysregulation, with repercussions on respiratory functions, or increasing the invasiveness of viral infection with associated lung injury. However, given that the interaction between influenza viruses and host cells exhibits unique characteristics, it is likely that the results obtained are influenced by the viral strain and the cell/in vivo model used in the experiments. Therefore, such kinds of experiments should be extended to a larger number of study models to obtain more comprehensive data.

Among the studies on host cellular factors here examined, those focusing on IL-36γ, TOMM34, and IRF5 [60,114,115] involved subjects with severe influenza-related symptoms. These studies have provided relevant insights into the molecular signature of influenza infection in humans, representing a useful premise for employing these factors as host biomarkers for the development of innovative laboratory diagnostic tools, broad-spectrum vaccines, and targeted treatments. Regarding the above-mentioned cellular determinants, to date, there is little scientific evidence about the availability of drugs and the effects induced by their depletion. Concerning IL-36γ, Tu et al. showed its inhibition by acitretin treatment in subjects with psoriasis [165], while Mizuno et al. focused on the effects of dexamethasone in the treatment of atopic dermatitis [166]. Interestingly, Gong et al. assessed that the depletion of microRNA-373 represses the replication of hepatitis C virus via activation of type 1 IFN response by targeting the cell factor IRF5 [167].

To investigate the clinical translational value of the information bearing on host cellular determinants, it is insightful to dissect their possible relationship with other respiratory viruses, such as respiratory syncytial virus (RSV) and severe acute respiratory syndrome coronavirus 2 (SARS-CoV-2), which have a significant impact during the influenza epidemic season in causing both single infections and co-infections with the influenza virus. In this regard, it is important to highlight that hypoxia-inducible factor-1α [168,169], interferon-induced protein 35 [117], Programmed Cell Death Protein 1/Programmed Cell Death Ligand 1 [170,171,172], and interferon regulator factor 5 [173,174] are also involved in severe infections caused by SARS-CoV-2. Moreover, other studies assessed the involvement of interleukin-6 [175,176,177,178,179,180] and suppressor of cytokine signaling-3 in both RSV and SARS-CoV-2 severe infections occurrence [181,182]. These data strengthen the information shown here, as they evidence that some cellular determinants are hijacked by different respiratory viruses, therefore representing a useful research starting point for the development of host-based preventive measures and treatments.

In recent years, increased attention has been paid to respiratory viral coinfections, thanks to the use of multiplex PCR in microbiological diagnosis. This technique may reveal the presence of multiple pathogens, but in general does not provide information on the predominant role of a single etiological agent in pathogenesis.

Although research advances have enabled the development of various experimental models, such as organoids, air–liquid interface cultures, and lung-on-a-chip platforms, to simulate the complexity of respiratory coinfections [183], little is known about the cellular biology of influenza virus superinfections and coinfections with RSV or SARS-CoV-2. It would be important to assess whether the use of cellular factors by these viruses during individual infections is altered by superinfection or coinfection, and what impact this has on disease severity.

Interestingly, Pinky et al. [184] demonstrated that the increased disease severity observed during murine IAV-RSV or IAV-SARS-CoV-2 coinfection was likely due to slower clearance of IAV-infected cells by the other viruses. Furthermore, the improved prognosis when IAV infection followed RSV infection could be reproduced when the clearance rate of RSV-infected cells was reduced by IAV. George et al. [185] demonstrated that RSV superinfection in IAV-infected mice is associated with higher IAV viral loads and increased morbidity and mortality. Shinjoh et al. [186] highlighted that although in vitro coinfection with IAV resulted in a reduction in RSV progeny, the extent of growth interference depended on the timing of IAV infection after RSV infection. Other authors [187] have shown that SARS-CoV-2 replication was promoted by the 2009 H1N1 IAV but hindered by adenovirus, suggesting the existence of different types of virus-virus interactions. Furthermore, superinfection experiments demonstrated that viral replication was influenced by the timing and multiplicity of infections used.

The above data attest to the highly complex nature of virus-virus interactions. Careful analysis of experimental results and clinical outcomes in cases of superinfection or co-infection is important to improve the prevention and treatment of respiratory infections, especially among high-risk individuals.

Although some homologies among the influenza virus-induced signaling pathways have been demonstrated, the mechanisms by which key host cell determinants are either singularly or synergistically involved remain unclear, particularly in high-risk populations. Therefore, in-depth studies integrating genomics, transcriptomics, proteomics, and metabolomics technologies are needed to enable researchers to better understand molecular mechanisms of influenza disease, identify host biomarkers, and predict therapeutic responses.

## 7. Conclusions

Preventing severe respiratory infections and enhancing diagnostic capabilities, particularly in a pandemic context, remains a major challenge, requiring an in-depth understanding of the molecular mechanisms of the disease pathology.

Here, numerous host factors implicated in the occurrence of severe influenza disease are discussed, offering a starting point for scientific research aimed at overcoming current limitations of vaccination and antiviral therapy and developing innovative medical approaches. It is noteworthy that some of the reported cellular factors are also exploited by RSV and SARS-CoV-2 to their advantage. Therefore, the future development of innovative vaccines and antiviral drugs based on host biomarkers could also be useful in cases of both single and co-infections caused by such viral agents.

Notably, evidencing the host risk factors involved in the progression to severe influenza may allow a more personalized medical approach to patient management, providing prompt diagnosis, risk assessment, and support for clinical decision-making. This knowledge should also help to develop innovative diagnostic tools based on host response molecular signatures.

## Figures and Tables

**Table 1:** Main genetic regulators of Interferon (IFN) responses.

Host Genetic Factors	Roles	References
Interferon Regulatory Factor (*IRF1*-*IRF9*)	Transcription factors involved in both induction and downstream signaling of the IFN system	[57,58,59,60,61,62,63,64,65,66,67,68]
Stimulator of Interferon Genes (STING) (*TMEM173*)	Adaptor protein mediating the responses to cytosolic DNA and leading to type I IFN production	[69,70,71,72]
Pattern recognition receptors (PRRs): - Toll-like receptors (TLRs) (*TLR1-TLR10*); - NOD-like receptors (NLRs) (*NOD1*, *NOD2*, *NLRP* subfamily, *NAIP*, *NLRX1*, and *CIITA*); - RIG-I-like receptors (RLRs) (RIGI/*DDX58*, *IFIH1*, and *DHX58*); - C-type lectin receptors (CLRs) (*Dectin-1* (*CLEC7A*) cluster and *Dectin-2* (*CLEC6A*) cluster)	Sensors that detect pathogens to stimulate IFN production	[73,74,75,76,77,78,79,80,81]

**Table 2:** Key host determinants linked to severe influenza.

Individual Subject Factors	Host Genetic Features	Host Cell Determinants
age 65 or older and less than two years	variants in genes that modulate the IFN-1 responses: *DDX58* (*RIGI*); *IFITM3*	factors enhancing influenza virus replication: Bcl-2-interacting killer (BIK) protein; hypoxia-inducible factor-1α (HIF-1α); importin-α7 protein; tetraspanin cluster of differentiation 151 (CD151)
chronic illnesses (diabetes; cardiovascular, renal, hematological, and pulmonary diseases)	variants in genes that heightened activation of the host immune response: *interleukin-1A* and *-1B*; *FCGR2A*; *C1QBP*; *TNF-α*	factors leading to dysregulation of immune system: interleukin 6 (IL-6); suppressor of cytokine signaling-3 (SOCS3); interleukin-36γ; translocase of the outer mitochondrial membrane 34 (TOMM34); eukaryotic translation initiation factor 48 (eIF48); interferon-induced protein 35 (IFP35); tripartite motif-containing 28 (TRIM28/KAP1/TIF1β); β6 integrin; tumor progression locus 2 (TPL2); acidic leucine-rich nuclear phosphoprotein 32B (ANP32B); PINK1/Parkin signaling pathway; programmed cell death protein 1/programmed cell death ligand 1 (PD-1/PD-L1) pathway; interferon regulator factor 5 (IRF5)
immunodeficiency	variants in genes playing a critical role in determining an individual’s susceptibility to severe disease: *CCR5*; *KIR*; *IGHG2*; *PTBP3*; *FTSJ3*; *CPVL*; *BST2*; *NOD*; *MAVS*; *CD55*	factors promoting the invasiveness of influenza viruses: cathepsin S (CTSS); mitochondrial transcription factor A (MTFA)
obesity	variants in genes that modulate influenza virus replication: *B3GALT5*; *ST6GAL1*	
bedridden condition		
pregnancy		

## Data Availability

No new data were created or analyzed in this study.

## Abbreviations

The following abbreviations are used in this manuscript:

ANP32	acidic leucine-rich nuclear phosphoprotein 32
BIK	Bcl-2-interacting killer
CD151	cluster of differentiation 151
CDC	Centers for Disease Control and Prevention
COVID-19	Coronavirus disease-19
CTSS	cathepsin S
HA	haemagglutinin
HIF	hypoxia-inducible factor 1
HK2	enzyme hexokinase 2
IAV	influenza A virus
IFITM3	interferon-induced transmembrane protein-3
IFP35	interferon-induced protein 35
INF	interferon
IRF5	interferon regulator factor 5
IL	interleukin
NA	neuraminidase
PD-1/PD-L1	programmed cell death protein 1/programmed cell death ligand 1
PTBP	polypyrimidine tract-binding protein
RSV	respiratory syncytial virus
SARS-CoV-2	severe acute respiratory syndrome coronavirus 2
SNP	single-nucleotide polymorphism
SOCS3	suppressor of cytokine signaling-3
TLR7	toll-like receptor 7
TNF	tumor necrosis factor-alpha
TOMM	translocase of the outer mitochondrial membrane
TPL2	tumor progression locus 2
TRIM28	tripartite motif-containing 28

## References

[1] Fereidouni S., Starick E., Karamendin K., Di Genova C., Scott S.D., Khan Y., Harder T., Kydyrmanov A. (2023). Genetic Characterization of a New Candidate Hemagglutinin Subtype of Influenza A Viruses. Emerg. Microbes Infect..

[2] Karakus U., Mena I., Kottur J., El Zahed S.S., Seoane R., Yildiz S., Chen L., Plancarte M., Lindsay L., Halpin R. (2024). H19 Influenza A Virus Exhibits Species-Specific MHC Class II Receptor Usage. Cell Host Microbe.

[3] Centers for Disease Control and Prevention Influenza (Flu). https://www.cdc.gov/flu/index.html.

[4] World Health Organization Influenza (Seasonal). https://www.who.int/news-room/fact-sheets/detail/influenza-(seasonal).

[5] Ackerson B., An J., Sy L.S., Solano Z., Slezak J., Tseng H.-F. (2020). Cost of Hospitalization Associated with Respiratory Syncytial Virus Infection Versus Influenza Infection in Hospitalized Older Adults. J. Infect. Dis..

[6] Haeberer M., López-Ibáñez de Aldecoa A., Seabroke S., Ramirez Agudelo J.L., Mora L., Sarabia L., Peerawaranun P., Meroc E., Aponte-Torres Z., Law A.W. (2025). Hospitalization Cost Estimates of Respiratory Syncytial Virus and Influenza Infections in Adults in Spain, 2016–2019. Vaccine.

[7] Mekkawy K., Abdalla F., Ali A. (2025). The Biology of Avian Influenza Virus: A Comprehensive Review with Insights into Novel Therapeutic Strategies. Open Vet. J..

[8] De Conto F. (2024). Avian Influenza A Viruses Modulate the Cellular Cytoskeleton during Infection of Mammalian Hosts. Pathogens.

[9] Centers for Disease Control and Prevention People at Increased Risk for Flu Complications. https://www.cdc.gov/flu/highrisk/index.htm.

[10] Beyer W.E.P., McElhaney J., Smith D.J., Monto A.S., Nguyen-Van-Tam J.S., Osterhaus A.D.M.E. (2013). Cochrane Re-Arranged: Support for Policies to Vaccinate Elderly People against Influenza. Vaccine.

[11] Remschmidt C., Wichmann O., Harder T. (2015). Vaccines for the Prevention of Seasonal Influenza in Patients with Diabetes: Systematic Review and Meta-Analysis. BMC Med..

[12] Remschmidt C., Wichmann O., Harder T. (2014). Influenza Vaccination in Patients with End-Stage Renal Disease: Systematic Review and Assessment of Quality of Evidence Related to Vaccine Efficacy, Effectiveness, and Safety. BMC Med..

[13] Sangkachai N., Gummow B., Hayakijkosol O., Suwanpakdee S., Wiratsudakul A. (2024). A Review of Risk Factors at the Human-Animal-Environmental Interface of Garbage Dumps That Are Driving Current and Emerging Zoonotic Diseases. One Health.

[14] Larsen S.V., Israelson R., Torp C., Larsen L.E., Jensen H.E., Kristensen C. (2025). Transmission, Pathological and Clinical Manifestations of Highly Pathogenic Avian Influenza A Virus in Mammals with Emphasis on H5N1 Clade 2.3.4.4b. Viruses.

[15] Santner P., Martins J.M.d.S., Kampmeyer C., Hartmann-Petersen R., Laursen J.S., Stein A., Olsen C.A., Arkin I.T., Winther J.R., Willemoës M. (2018). Random Mutagenesis Analysis of the Influenza A M2 Proton Channel Reveals Novel Resistance Mutants. Biochemistry.

[16] Cheung I.M.Y., Paynter J., Broderick D., Trenholme A., Byrnes C.A., Grant C.C., Huang S.Q., Turner N., McIntyre P. (2024). Severe Acute Respiratory Infection (SARI) Due to Influenza in Post-COVID Resurgence: Disproportionate Impact on Older Māori and Pacific Peoples. Influenza Other Respir. Viruses.

[17] Khieu T.Q.T., Pierse N., Telfar-Barnard L.F., Zhang J., Huang Q.S., Baker M.G. (2017). Modelled Seasonal Influenza Mortality Shows Marked Differences in Risk by Age, Sex, Ethnicity and Socioeconomic Position in New Zealand. J. Infect..

[18] Bosaeed M., Kumar D. (2018). Seasonal Influenza Vaccine in Immunocompromised Persons. Hum. Vaccin. Immunother..

[19] Nguyen O.N., Garzón-Orjuela N., van der Velden A.W., Butler C.C., Vellinga A. (2025). Obesity in Recovery from Influenza-like Illness and Effectiveness of Oseltamivir. Influenza Other Respir. Viruses.

[20] Noye E.C., Bekkering S., Sng J.D.J., Burgner D., Longmore D.K., Short K.R. (2025). Obesity Is a Risk Factor for Severe Influenza Virus Infection and COVID-19 in Children. J. Pediatr. Infect. Dis. Soc..

[21] Honce R., Schultz-Cherry S. (2019). Impact of Obesity on Influenza A Virus Pathogenesis, Immune Response, and Evolution. Front. Immunol..

[22] Hulme K.D., Noye E.C., Short K.R., Labzin L.I. (2021). Dysregulated Inflammation During Obesity: Driving Disease Severity in Influenza Virus and SARS-CoV-2 Infections. Front. Immunol..

[23] Namkoong H., Ishii M., Fujii H., Asami T., Yagi K., Suzuki S., Azekawa S., Tasaka S., Hasegawa N., Betsuyaku T. (2019). Obesity Worsens the Outcome of Influenza Virus Infection Associated with Impaired Type I Interferon Induction in Mice. Biochem. Biophys. Res. Commun..

[24] Alarcon P.C., Ulanowicz C.J., Damen M.S.M.A., Eom J., Sawada K., Chung H., Alahakoon T., Oates J.R., Wayland J.L., Stankiewicz T.E. (2025). Obesity Uncovers the Presence of Inflammatory Lung Macrophage Subsets with an Adipose Tissue Transcriptomic Signature in Influenza Virus Infection. J. Infect. Dis..

[25] Radigan K.A., Morales-Nebreda L., Soberanes S., Nicholson T., Nigdelioglu R., Cho T., Chi M., Hamanaka R.B., Misharin A.V., Perlman H. (2014). Impaired Clearance of Influenza A Virus in Obese, Leptin Receptor Deficient Mice Is Independent of Leptin Signaling in the Lung Epithelium and Macrophages. PLoS ONE.

[26] Milner J.J., Rebeles J., Dhungana S., Stewart D.A., Sumner S.C.J., Meyers M.H., Mancuso P., Beck M.A. (2015). Obesity Increases Mortality and Modulates the Lung Metabolome during Pandemic H1N1 Influenza Virus Infection in Mice. J. Immunol..

[27] Knoll M., Honce R., Meliopoulos V., Segredo-Otero E.A., Johnson K.E.E., Schultz-Cherry S., Ghedin E., Gresham D. (2024). Host Obesity Impacts Genetic Variation in Influenza A Viral Populations. J. Virol..

[28] Famati E.A., Ujamaa D., O’Halloran A., Kirley P.D., Chai S.J., Armistead I., Alden N.B., Yousey-Hindes K., Openo K.P., Ryan P.A. (2023). Association of Chronic Medical Conditions with Severe Outcomes Among Nonpregnant Adults 18–49 Years Old Hospitalized with Influenza, FluSurv-NET, 2011–2019. Open Forum Infect. Dis..

[29] Nakashima H., Fujiwara S., Honda S., Tominaga R., Yokoyama D., Noguchi A., Furuki S., Koyama S., Murahashi R., Ikeda T. (2025). Risk Factors for Influenza Virus Infection in Patients with Hematological Disease. Leuk. Lymphoma.

[30] Andrew M.K., Pott H., Staadegaard L., Paget J., Chaves S.S., Ortiz J.R., McCauley J., Bresee J., Nunes M.C., Baumeister E. (2023). Age Differences in Comorbidities, Presenting Symptoms, and Outcomes of Influenza Illness Requiring Hospitalization: A Worldwide Perspective from the Global Influenza Hospital Surveillance Network. Open Forum Infect. Dis..

[31] Harada K., Onizuka M., Mori T., Shimizu H., Seo S., Aotsuka N., Takeda Y., Sekiya N., Kusuda M., Fujiwara S. (2023). Prognostic Factors for the Development of Lower Respiratory Tract Infection after Influenza Virus Infection in Allogeneic Hematopoietic Stem Cell Transplantation Recipients: A Kanto Study Group for Cell Therapy Multicenter Analysis. Int. J. Infect. Dis..

[32] Lapinsky S.E. (2010). H1N1 Novel Influenza A in Pregnant and Immunocompromised Patients. Crit. Care Med..

[33] Xing Y., Bahl A. (2025). Comparative Analysis of Severe Clinical Outcomes in Hospitalized Patients with RSV, Influenza, and COVID-19 Across Early and Late COVID-19 Pandemic Phases (2021–2024). J. Clin. Med..

[34] Chen L., Han X., Li Y., Zhang C., Xing X. (2021). The Severity and Risk Factors for Mortality in Immunocompromised Adult Patients Hospitalized with Influenza-Related Pneumonia. Ann. Clin. Microbiol. Antimicrob..

[35] Nichols W.G., Guthrie K.A., Corey L., Boeckh M. (2004). Influenza Infections after Hematopoietic Stem Cell Transplantation: Risk Factors, Mortality, and the Effect of Antiviral Therapy. Clin. Infect. Dis..

[36] Lin J.C., Nichol K.L. (2001). Excess Mortality Due to Pneumonia or Influenza During Influenza Seasons Among Persons with Acquired Immunodeficiency Syndrome. Arch. Intern. Med..

[37] Fica A., Pinto F., Sotomayor V., Fasce R., Andrade W., Dabanch J., Soto A., Triantafilo V. (2019). Host Characteristics Predict Outcome among Adult Patients Admitted by Severe Acute Respiratory Infection. Rev. Med. Chil..

[38] Nakajoh K., Nakagawa T., Sekizawa K., Matsui T., Arai H., Sasaki H. (2000). Relation between Incidence of Pneumonia and Protective Reflexes in Post-stroke Patients with Oral or Tube Feeding. J. Intern. Med..

[39] Carr E.J., Dooley J., Garcia-Perez J.E., Lagou V., Lee J.C., Wouters C., Meyts I., Goris A., Boeckxstaens G., Linterman M.A. (2016). The Cellular Composition of the Human Immune System Is Shaped by Age and Cohabitation. Nat. Immunol..

[40] Mai K., Pan W., Lin Z., Wang Y., Yang Z. (2025). Pathogenesis of Influenza and SARS-CoV-2 Co-Infection at the Extremes of Age: Decipher the Ominous Tales of Immune Vulnerability. Adv. Biotechnol..

[41] Kasmani M.Y., Topchyan P., Brown A.K., Brown R.J., Wu X., Chen Y., Khatun A., Alson D., Wu Y., Burns R. (2023). A Spatial Sequencing Atlas of Age-Induced Changes in the Lung during Influenza Infection. Nat. Commun..

[42] Samy R.P., Lim L.H.K. (2015). DAMPs and Influenza Virus Infection in Ageing. Ageing Res. Rev..

[43] Gill P.J., Ashdown H.F., Wang K., Heneghan C., Roberts N.W., Harnden A., Mallett S. (2015). Identification of Children at Risk of Influenza-Related Complications in Primary and Ambulatory Care: A Systematic Review and Meta-Analysis. Lancet Respir. Med..

[44] Park J.Y., Dowell A., Turner N., Albrecht S., Hills I., Marsh S. (2025). Risk and Resilience Factors Associated with the Progression of Influenza to Severe Disease Outcomes: Umbrella Review Protocol. BMJ Open.

[45] Yang S., Lu S., Qi C., Guo Y., Wang L. (2025). Risk Factors for Re-Hospitalization within 90 Days of Discharge for Severe Influenza in Children. BMC Infect. Dis..

[46] Hernandez N., Melki I., Jing H., Habib T., Huang S.S.Y., Danielson J., Kula T., Drutman S., Belkaya S., Rattina V. (2018). Life-Threatening Influenza Pneumonitis in a Child with Inherited IRF9 Deficiency. J. Exp. Med..

[47] Metcalf C.J., Chen S., García-Carreras B., Hay J., Zhu H., Jiang C., Kwok K., Riley S., Read J., Lessler J. (2025). Sex Differences in Antibody Responses to Influenza A/H3N2 across the Life Course. Res. Sq..

[48] Cervantes-Gonzalez M., Launay O. (2010). Pandemic Influenza A (H1N1) in Pregnant Women: Impact of Early Diagnosis and Antiviral Treatment. Expert Rev. Anti. Infect. Ther..

[49] Siston A.M. (2010). Pandemic 2009 Influenza A(H1N1) Virus Illness Among Pregnant Women in the United States. JAMA.

[50] Vanders R.L., Gibson P.G., Wark P.A.B., Murphy V.E. (2013). Alterations in Inflammatory, Antiviral and Regulatory Cytokine Responses in Peripheral Blood Mononuclear Cells from Pregnant Women with Asthma. Respirology.

[51] Zheng R., Qin X., Li Y., Yu X., Wang J., Tan M., Yang Z., Li W. (2012). Imbalanced Anti-H1N1 Immunoglobulin Subclasses and Dysregulated Cytokines in Hospitalized Pregnant Women with 2009 H1N1 Influenza and Pneumonia in Shenyang, China. Hum. Immunol..

[52] Forbes R.L., Wark P.A.B., Murphy V.E., Gibson P.G. (2012). Pregnant Women Have Attenuated Innate Interferon Responses to 2009 Pandemic Influenza A Virus Subtype H1N1. J. Infect. Dis..

[53] Vanders R.L., Gibson P.G., Murphy V.E., Wark P.A.B. (2013). Plasmacytoid Dendritic Cells and CD8 T Cells from Pregnant Women Show Altered Phenotype and Function Following H1N1/09 Infection. J. Infect. Dis..

[54] Valenzuela-Sánchez F., Valenzuela-Méndez B., Rodríguez-Gutiérrez J.F., Rello J. (2016). Personalized Medicine in Severe Influenza. Eur. J. Clin. Microbiol. Infect. Dis..

[55] Noah D.L., Noah J.W. (2013). Adapting Global Influenza Management Strategies to Address Emerging Viruses. Am. J. Physiol. Lung Cell Mol. Physiol..

[56] Schmidt A., Groh A.M., Frick J.S., Vehreschild M.J.G.T., Ludwig K.U. (2022). Genetic Predisposition and the Variable Course of Infectious Diseases. Dtsch. Arztebl. Int..

[57] Cui J., Foo S.S.W., Kong W.T., Lin C., Ampomah P.B., Zharkova O., Chai L.S., Sachaphibulkij K., Arora S., Kaliaperumal N. (2026). *Irf7* Deficiency Confers Protection Against Influenza Infection, Independent of *Irf3*. Int. J. Biol. Sci..

[58] Zou X., Xiang X., Chen Y., Peng T., Luo X., Pan Z. (2010). Understanding Inhibition of Viral Proteins on Type I IFN Signaling Pathways with Modeling and Optimization. J. Theor. Biol..

[59] Chen X., Guan Y., Li K., Luo T., Mu Y., Chen X. (2021). IRF1 and IRF2 Act as Positive Regulators in Antiviral Response of Large Yellow Croaker (*Larimichthys crocea*) by Induction of Distinct Subgroups of Type I IFNs. Dev. Comp. Immunol..

[60] Wang X., Guo J., Wang Y., Xiao Y., Wang L., Hua S. (2018). Genetic Variants of Interferon Regulatory Factor 5 Associated with the Risk of Community-Acquired Pneumonia. Gene.

[61] Sy B.T., Hoan N.X., Van Tong H., Meyer C.G., Toan N.L., Song L.H., Bock C.-T., Velavan T.P. (2018). Genetic Variants of Interferon Regulatory Factor 5 Associated with Chronic Hepatitis B Infection. World J. Gastroenterol..

[62] Lin Z., Wang J., Zhao S., Li Y., Zhang Y., Wang Y., Yan Y., Cheng Y., Sun J. (2022). Goose IRF7 Is Involved in Antivirus Innate Immunity by Mediating IFN Activation. Dev. Comp. Immunol..

[63] Kanno T., Miyako K., Nakajima T., Yokoyama S., Sasamoto S., Asou H.K., Ohara O., Nakayama T., Endo Y. (2022). SCD2-Mediated Cooperative Activation of IRF3-IRF9 Regulatory Circuit Controls Type I Interferon Transcriptome in CD4^+^ T Cells. Front. Immunol..

[64] Li L., Fu J., Li J., Guo S., Chen Q., Zhang Y., Liu Z., Tan C., Chen H., Wang X. (2022). African Swine Fever Virus PI215L Inhibits Type I Interferon Signaling by Targeting Interferon Regulatory Factor 9 for Autophagic Degradation. J. Virol..

[65] Talaat R.M., Elsayed S.S., Abdel-Hakem N.E., El-Shenawy S.Z. (2022). Genetic Polymorphism in Toll-like Receptor 3 and Interferon Regulatory Factor 3 in Hepatitis C Virus-Infected Patients: Correlation with Liver Cirrhosis. Viral Immunol..

[66] Costa A.S., Agostini S., Guerini F.R., Mancuso R., Zanzottera M., Ripamonti E., Racca V., Nemni R., Clerici M. (2017). Modulation of Immune Responses to Herpes Simplex Virus Type 1 by IFNL3 and IRF7 Polymorphisms: A Study in Alzheimer’s Disease. J. Alzheimer’s Dis..

[67] Real L.M., Caruz A., Rivero-Juarez A., Soriano V., Neukam K., Rivero A., Cifuentes C., Mira J.A., Macías J., Pineda J.A. (2014). A Polymorphism Linked to RRAS, SCAF1, IRF3 and BCL2L12 Genes Is Associated with Cirrhosis in Hepatitis C Virus Carriers. Liver Int..

[68] Chen Y., Yang H., Wu X., Liu Z., Chen Y., Wei Q., Lin J., Yu Y., Tu Q., Li H. (2024). Interferon Regulatory Factors (*IRF1*, *IRF4*, *IRF5*, *IRF7* and *IRF9*) in Sichuan taimen (*Hucho bleekeri*): Identification and Functional Characterization. Genes.

[69] Taguchi T. (2026). STING Innate Immunity Signalling from the Golgi. Subcell. Biochem..

[70] Mashkovskaia A., Agapkina Y., Oretskaya T., Gottikh M., Anisenko A. (2025). HIV-1 and Its Strategy for Hiding Viral CDNA from STING-Mediated Innate Immunity. Int. J. Mol. Sci..

[71] Ye R., Wang S., Hu Y., Pan Y., Zheng W., Xia F., Wang Y., Guo H., Zheng S., Wei W. (2026). STING–NF-ΚB Signaling Builds an Influenza Spillover Barrier. Science (1979).

[72] Zhu Q., Yu S. (2025). The Role of CGAS-STING Signaling in HPV Infection and HPV-Related Cancers. Front. Immunol..

[73] Sæterhaug Bye K., Rian K., Ryan L., Espevik T., Anthonsen M.W., Yurchenko M. (2025). The Immune Receptors TLR4 and SLAMF1 Regulate TNF Release by Human Metapneumovirus in Human Macrophages. Front. Immunol..

[74] Zahmatkesh A., Yaghobi R., Sahragard I., Afshari A., Hosseini S.Y., Kargar M. (2025). Measuring MRNA Expression Level of Viral Genes, IRF3/7 and TLR7/8 during BK Polyomavirus Infection in Kidney Transplant Recipients. Immunobiology.

[75] Brunner M.B., Rosales J.J., Ladera M., Nieto Farías M.V., Verna A., Pérez S. (2025). TLR3 Regulation and Cytokine Response during BoAHV-1 and BoAHV-5 Infection of Neuronal-like Cells. Microbes Infect..

[76] Hromić-Jahjefendić A., Aljabali A.A.A. (2025). Analysis of the Immune Response in COVID-19. Prog. Mol. Biol. Transl. Sci..

[77] Jiao Q., Zhu S., Liao B., Liu H., Guo X., Wu L., Chen C., Peng L., Xie C. (2024). An NLR Family Member X1 Mutation (p.Arg707Cys) Suppresses Hepatitis B Virus Infection in Hepatocytes and Favors the Interaction of Retinoic Acid-Inducible Gene 1 with Mitochondrial Antiviral Signaling Protein. Arch. Virol..

[78] Ohto U. (2022). Activation and Regulation Mechanisms of NOD-like Receptors Based on Structural Biology. Front. Immunol..

[79] Domínguez-Martínez D.A., Pérez-Flores M.S., Núñez-Avellaneda D., Torres-Flores J.M., León-Avila G., García-Pérez B.E., Salazar M.I. (2024). NOD2 Responds to Dengue Virus Type 2 Infection in Macrophage-like Cells Interacting with MAVS Adaptor and Affecting IFN-α Production and Virus Titers. Pathogens.

[80] van Huizen M., Gack M.U. (2025). The RIG-I-like Receptor Family of Immune Proteins. Mol. Cell.

[81] Isazadeh A., Heris J.A., Shahabi P., Mohammadinasab R., Shomali N., Nasiri H., Valedkarimi Z., Khosroshahi A.J., Hajazimian S., Akbari M. (2023). Pattern-Recognition Receptors (PRRs) in SARS-CoV-2. Life Sci..

[82] Ciancanelli M.J., Huang S.X.L., Luthra P., Garner H., Itan Y., Volpi S., Lafaille F.G., Trouillet C., Schmolke M., Albrecht R.A. (2015). Life-Threatening Influenza and Impaired Interferon Amplification in Human IRF7 Deficiency. Science (1979).

[83] Lim H.K., Huang S.X.L., Chen J., Kerner G., Gilliaux O., Bastard P., Dobbs K., Hernandez N., Goudin N., Hasek M.L. (2019). Severe Influenza Pneumonitis in Children with Inherited TLR3 Deficiency. J. Exp. Med..

[84] Lee S., Zhang Y., Newhams M., Novak T., Thomas P.G., Mourani P.M., Hall M.W., Loftis L.L., Cvijanovich N.Z., Tarquinio K.M. (2022). *DDX58* Is Associated with Susceptibility to Severe Influenza Virus Infection in Children and Adolescents. J. Infect. Dis..

[85] Choudhary M.L., Chaudhary U., Salve M., Shinde P., Padbidri V., Sangle S.A., Salvi S., Bavdekar A.R., D’costa P., Alagarasu K. (2022). Functional Single-Nucleotide Polymorphisms in the *MBL2* and *TLR3* Genes Influence Disease Severity in Influenza A (H1N1)Pdm09 Virus-Infected Patients from Maharashtra, India. Viral Immunol..

[86] Yang X., Tan B., Zhou X., Xue J., Zhang X., Wang P., Shao C., Li Y., Li C., Xia H. (2015). Interferon-Inducible Transmembrane Protein 3 Genetic Variant Rs12252 and Influenza Susceptibility and Severity: A Meta-Analysis. PLoS ONE.

[87] Pan Y., Yang P., Dong T., Zhang Y., Shi W., Peng X., Cui S., Zhang D., Lu G., Liu Y. (2017). *IFITM3* Rs12252-C Variant Increases Potential Risk for Severe Influenza Virus Infection in Chinese Population. Front. Cell. Infect. Microbiol..

[88] Denz P.J., Yount J.S. (2025). *IFITM3* Variants Point to a Critical Role in Emergent Virus Infections. mBio.

[89] Everitt A.R., Clare S., Pertel T., John S.P., Wash R.S., Smith S.E., Chin C.R., Feeley E.M., Sims J.S., Adams D.J. (2012). *IFITM3* Restricts the Morbidity and Mortality Associated with Influenza. Nature.

[90] Allen E.K., Randolph A.G., Bhangale T., Dogra P., Ohlson M., Oshansky C.M., Zamora A.E., Shannon J.P., Finkelstein D., Dressen A. (2017). SNP-Mediated Disruption of CTCF Binding at the *IFITM3* Promoter Is Associated with Risk of Severe Influenza in Humans. Nat. Med..

[91] Jia R., Pan Q., Ding S., Rong L., Liu S.-L., Geng Y., Qiao W., Liang C. (2012). The N-Terminal Region of *IFITM3* Modulates Its Antiviral Activity by Regulating *IFITM3* Cellular Localization. J. Virol..

[92] Jia R., Xu F., Qian J., Yao Y., Miao C., Zheng Y.M., Liu S.L., Guo F., Geng Y., Qiao W. (2014). Identification of an Endocytic Signal Essential for the Antiviral Action of *IFITM3*. Cell. Microbiol..

[93] Denz P.J., Speaks S., Kenney A.D., Eddy A.C., Papa J.L., Roettger J., Scace S.C., Hemann E.A., Forero A., Webby R.J. (2023). Innate immune control of influenza virus interspecies adaptation via IFITM3. Nat. Commun..

[94] Liu Y., Li S., Zhang G., Nie G., Meng Z., Mao D., Chen C., Chen X., Zhou B., Zeng G. (2013). Genetic Variants in IL1A and IL1B Contribute to the Susceptibility to 2009 Pandemic H1N1 Influenza A Virus. BMC Immunol..

[95] Zúñiga J., Buendía-Roldán I., Zhao Y., Jiménez L., Torres D., Romo J., Ramírez G., Cruz A., Vargas-Alarcon G., Sheu C.C. (2012). Genetic Variants Associated with Severe Pneumonia in A/H1N1 Influenza Infection. Eur. Respir. J..

[96] García-Ramírez R.A., Ramírez-Venegas A., Quintana-Carrillo R., Camarena A.E., Falfán-Valencia R., Mejía-Aranguré J.M. (2015). *TNF*, *IL6*, and *IL1B* Polymorphisms are Associated with Severe Influenza a (H1N1) Virus Infection in the Mexican Population. PLoS ONE.

[97] Keynan Y., Malik S., Fowke K.R. (2013). The Role of Polymorphisms in Host Immune Genes in Determining the Severity of Respiratory Illness Caused by Pandemic H1N1 Influenza. Public Health Genom..

[98] Falcon A., Cuevas M.T., Rodriguez-Frandsen A., Reyes N., Pozo F., Moreno S., Ledesma J., Martínez-Alarcón J., Nieto A., Casas I. (2015). CCR5 Deficiency Predisposes to Fatal Outcome in Influenza Virus Infection. J. Gen. Virol..

[99] Li M., Chen Y., Chen T., Hu S., Chen L., Shen L., Li F., Yang J., Sun Y., Wang D. (2021). A Host-Based Whole Genome Sequencing Study Reveals Novel Risk Loci Associated with Severity of Influenza A(H1N1)Pdm09 Infection. Emerg. Microbes Infect..

[100] Chatzopoulou F., Gioula G., Kioumis I., Chatzidimitriou D., Exindari M. (2019). Identification of Complement-Related Host Genetic Risk Factors Associated with Influenza A(H1N1)Pdm09 Outcome: Challenges Ahead. Med. Microbiol. Immunol..

[101] Kosmicki J.A., Marcketta A., Sharma D., Di Gioia S.A., Batista S., Yang X.M., Tzoneva G., Martinez H., Sidore C., Kessler M.D. (2024). Genetic Risk Factors for COVID-19 and Influenza Are Largely Distinct. Nat. Genet..

[102] Kim Y.-C., Jeong B.-H. (2020). Ethnic Variation in Risk Genotypes Based on Single Nucleotide Polymorphisms (SNPs) of the Interferon-Inducible Transmembrane 3 (*IFITM3*) Gene, a Susceptibility Factor for Pandemic 2009 H1N1 Influenza A Virus. Immunogenetics.

[103] Hui X., Tian X., Ding S., Gao G., Zhao X., Cui J., Hou Y., Zhao T., Wang H. (2025). Metabolic Hostile Takeover: How Influenza Virus Reprograms Cellular Metabolism for Replication. Viruses.

[104] Liu C., Ma Q., Yang Y., Rong R. (2026). Metabolomics in Influenza Viral Infection: Insights into Host–Virus Interactions and Potential Biomarkers of Severe Outcomes. Crit. Rev. Microbiol..

[105] Soni S., Yildiz S., Allen E.K., Petersen H., Peeples M., El Zahed S., Rosas L., Anang V., Antonescu L., Nho R.S. (2025). BIK Polymorphism and Proteasome Regulation Unveil Host Risk Factor for Severe Influenza. Proc. Natl. Acad. Sci. USA.

[106] Soni S., Mebratu Y.A. (2025). Polymorphism of BIK as a Host Risk Factor for Severe Influenza. DNA Cell Biol..

[107] Meng X., Zhu Y., Yang W., Zhang J., Jin W., Tian R., Yang Z., Wang R. (2024). HIF-1α Promotes Virus Replication and Cytokine Storm in H1N1 Virus-Induced Severe Pneumonia through Cellular Metabolic Reprogramming. Virol. Sin..

[108] Resa-Infante P., Thieme R., Ernst T., Arck P.C., Ittrich H., Reimer R., Gabriel G. (2014). Importin-A7 Is Required for Enhanced Influenza A Virus Replication in the Alveolar Epithelium and Severe Lung Damage in Mice. J. Virol..

[109] Wong A.H., Tran T. (2020). CD151 in Respiratory Diseases. Front. Cell Dev. Biol..

[110] Qiao Y., Yan Y., Tan K.S., Tan S.S.L., Seet J.E., Arumugam T.V., Chow V.T.K., Wang D.Y., Tran T. (2018). CD151, a Novel Host Factor of Nuclear Export Signaling in Influenza Virus Infection. J. Allergy Clin. Immunol..

[111] Li X., Huang C., Rai K.R., Xu Q. (2025). Innate Immune Role of IL-6 in Influenza a Virus Pathogenesis. Front. Cell. Infect. Microbiol..

[112] Liu S., Qiu F., Gu R., Xu E. (2024). Functional Involvement of Signal Transducers and Activators of Transcription in the Pathogenesis of Influenza A Virus. Int. J. Mol. Sci..

[113] Liu S., Yan R., Chen B., Pan Q., Chen Y., Hong J., Zhang L., Liu W., Wang S., Chen J.-L. (2019). Influenza Virus-Induced Robust Expression of SOCS3 Contributes to Excessive Production of IL-6. Front. Immunol..

[114] Liu S., Li H., Wang Y., Li H., Du S., Zou X., Zhang X., Cao B. (2020). High Expression of IL-36γ in Influenza Patients Regulates Interferon Signaling Pathway and Causes Programmed Cell Death During Influenza Virus Infection. Front. Immunol..

[115] Shi Q., Zhang P., Hu Q., Zhang T., Hou R., Yin S., Zou Y., Chen F., Jiao S., Si L. (2024). Role of TOMM34 on NF-ΚB Activation-Related Hyperinflammation in Severely Ill Patients with COVID-19 and Influenza. EBioMedicine.

[116] Wang S., Chi X., Wei H., Chen Y., Chen Z., Huang S., Chen J.-L. (2014). Influenza A Virus-Induced Degradation of Eukaryotic Translation Initiation Factor 4B Contributes to Viral Replication by Suppressing *IFITM3* Protein Expression. J. Virol..

[117] Yu Y., Xu N., Cheng Q., Deng F., Liu M., Zhu A., Min Y.-Q., Zhu D., Huang W., Feng X. (2021). IFP35 as a Promising Biomarker and Therapeutic Target for the Syndromes Induced by SARS-CoV-2 or Influenza Virus. Cell Rep..

[118] Yang S., Adaway M., Du J., Huang S., Sun J., Bidwell J.P., Zhou B. (2021). NMP4 Regulates the Innate Immune Response to Influenza A Virus Infection. Mucosal Immunol..

[119] Krischuns T., Günl F., Henschel L., Binder M., Willemsen J., Schloer S., Rescher U., Gerlt V., Zimmer G., Nordhoff C. (2018). Phosphorylation of TRIM28 Enhances the Expression of IFN-β and Proinflammatory Cytokines During HPAIV Infection of Human Lung Epithelial Cells. Front. Immunol..

[120] Meliopoulos V., Livingston B., Van de Velde L.-A., Honce R., Schultz-Cherry S. (2019). Absence of Β6 Integrin Reduces Influenza Disease Severity in Highly Susceptible Obese Mice. J. Virol..

[121] Smith M., Meliopoulos V., Tan S., Bub T., Brigleb P.H., Sharp B., Crawford J.C., Prater M.S., Pruett-Miller S.M., Schultz-Cherry S. (2023). The Β6 Integrin Negatively Regulates TLR7-Mediated Epithelial Immunity via Autophagy During Influenza A Virus Infection. bioRxiv.

[122] Latha K., Jamison K.F., Watford W.T. (2021). Tpl2 Ablation Leads to Hypercytokinemia and Excessive Cellular Infiltration to the Lungs During Late Stages of Influenza Infection. Front. Immunol..

[123] Latha K., Rao S., Sakamoto K., Watford W.T. (2022). Tumor Progression Locus 2 Protects against Acute Respiratory Distress Syndrome in Influenza A Virus-Infected Mice. Microbiol. Spectr..

[124] Latha K., Patel Y., Rao S., Watford W.T. (2023). The Influenza-Induced Pulmonary Inflammatory Exudate in Susceptible Tpl2-Deficient Mice Is Dictated by Type I IFN Signaling. Inflammation.

[125] Beck S., Zickler M., Pinho dos Reis V., Günther T., Grundhoff A., Reilly P.T., Mak T.W., Stanelle-Bertram S., Gabriel G. (2020). ANP32B Deficiency Protects Mice from Lethal Influenza A Virus Challenge by Dampening the Host Immune Response. Front. Immunol..

[126] Staller E., Carrique L., Swann O.C., Fan H., Keown J.R., Sheppard C.M., Barclay W.S., Grimes J.M., Fodor E. (2024). Structures of H5N1 Influenza Polymerase with ANP32B Reveal Mechanisms of Genome Replication and Host Adaptation. Nat. Commun..

[127] Huo C., Li Y., Tang Y., Su R., Xu J., Dong H., Hu Y., Yang H. (2025). Vital Role of PINK1/Parkin-Mediated Mitophagy of Pulmonary Epithelial Cells in Severe Pneumonia Induced by IAV and Secondary Staphylococcus Aureus Infection. Int. J. Mol. Sci..

[128] Jiang Z., Pan W., Chen Y., Zhou D., Ren S., Tong Q., Liu L., Sun H., Sun Y., Bi Y. (2025). ApoD Mediates Age-Associated Increase in Vulnerability to Influenza Virus Infection. Proc. Natl. Acad. Sci. USA.

[129] Ou H., Chen L., Wu H. (2022). Enhanced Programmed Cell Death Protein 1/Programmed Cell Death Ligand 1 Expression Induced by Severe Influenza A Virus Infection Impairs Host’s Antiviral Response. Viral Immunol..

[130] Rutigliano J.A., Sharma S., Morris M.Y., Oguin T.H., McClaren J.L., Doherty P.C., Thomas P.G. (2014). Highly Pathological Influenza A Virus Infection Is Associated with Augmented Expression of PD-1 by Functionally Compromised Virus-Specific CD8^+^ T Cells. J. Virol..

[131] Forbester J.L., Clement M., Wellington D., Yeung A., Dimonte S., Marsden M., Chapman L., Coomber E.L., Tolley C., Lees E. (2020). IRF5 Promotes Influenza Virus-Induced Inflammatory Responses in Human Induced Pluripotent Stem Cell-Derived Myeloid Cells and Murine Models. J. Virol..

[132] Wang X., Guo J., Wang Y., Xiao Y., Wang L., Hua S. (2018). Expression Levels of Interferon Regulatory Factor 5 (IRF5) and Related Inflammatory Cytokines Associated with Severity, Prognosis, and Causative Pathogen in Patients with Community-Acquired Pneumonia. Med. Sci. Monit..

[133] Heindel D.W., Koppolu S., Zhang Y., Kasper B., Meche L., Vaiana C.A., Bissel S.J., Carter C.E., Kelvin A.A., Elaish M. (2020). Glycomic Analysis of Host Response Reveals High Mannose as a Key Mediator of Influenza Severity. Proc. Natl. Acad. Sci. USA.

[134] Ma T., Yang C., Wang Y., Tu C., Zhang J., Mai K., Wu S., Zhou H., Li S., Ye S. (2025). Cathepsin S Contributes to Influenza-Induced Lung Injury by Driving Inflammation, Promoting Apoptosis, and Disrupting Epithelial Barrier Integrity. Microbiol. Spectr..

[135] Gao X., Zhu B., Wu Y., Li C., Zhou X., Tang J., Sun J. (2022). TFAM-Dependent Mitochondrial Metabolism Is Required for Alveolar Macrophage Maintenance and Homeostasis. J. Immunol..

[136] Dapat C., Peck H., Jelley L., Diefenbach-Elstob T., Slater T., Hussain S., Britton P., Cheng A.C., Wood T., Howard-Jones A. (2025). Extended Influenza Seasons in Australia and New Zealand in 2025 Due to the Emergence of Influenza A(H3N2) Subclade K Viruses. Eurosurveillance.

[137] European Centre for Disease Prevention and Control Early Estimates of Seasonal Influenza Vaccine Effectiveness Against Influenza Requiring Medical Attention at Primary Care Level in Europe, Week 41–49, 2025. https://www.ecdc.europa.eu/en/news-events/early-estimates-seasonal-influenza-vaccine-effectiveness-against-influenza-requiring.

[138] World Health Organization More than Half of WHO European Region Experiencing Intense, Early Influenza Sea-Son Driven by New Strain. https://www.who.int/europe/news/item/17-12-2025-more-than-half-of-who-european-region-experiencing-intense--early-influenza-season-driven-by-new-strain.

[139] Centers for Disease Control and Prevention Weekly US Influenza Surveillance Report: Key Updates for Week 3, Ending January 24, 2026. https://www.cdc.gov/fluview/surveillance/2026-week-03.html.

[140] World Health Organization (2026). Global Respiratory Virus Activity: Weekly Update N° 561. https://www.who.int/publications/m/item/global-respiratory-virus-activity--weekly-update-n--561.

[141] Cheng Y., Cao X., Cao Z., Xu C., Sun L., Gao Y., Wang Y., Li S., Wu C., Li X. (2020). Effects of Influenza Vaccination on the Risk of Cardiovascular and Respiratory Diseases and All-Cause Mortality. Ageing Res. Rev..

[142] Nuwarda R.F., Alharbi A.A., Kayser V. (2021). An Overview of Influenza Viruses and Vaccines. Vaccines.

[143] Krauland M.G., Mandell A., Roberts M.S. (2025). Estimated Burden of Influenza and Direct and Indirect Benefits of Influenza Vaccination. JAMA Netw. Open.

[144] Grohskopf L.A., Blanton L.H., Ferdinands J.M., Reed C., Dugan V.G., Daskalakis D.C. (2025). Prevention and Control of Seasonal Influenza with Vaccines: Recommendations of the Advisory Committee on Immunization Practices—United States, 2025–2026 Influenza Season. MMWR Morb. Mortal. Wkly. Rep..

[145] Boccalini S., de Waure C., Martorella L., Orlando P., Bonanni P., Bechini A. (2025). The Evolution of Annual Immunization Recommendations Against Influenza in Italy: The Path to Precision Vaccination. Vaccines.

[146] Leonforte F., Fiorilla C., Giorgianni G., Nicosia V., Contarino F., Genovese C., Genovese G., Morlino G., Chimienti M., Mistretta A. (2025). Influenza Vaccination Appropriateness: Insights from the Local Health Unit of Catania During the 2023/2024 and 2024/2025 Seasons. Vaccines.

[147] Dubé E., Laberge C., Guay M., Bramadat P., Roy R., Bettinger J.A. (2013). Vaccine Hesitancy. Hum. Vaccin. Immunother..

[148] World Health Organization Ten Threats to Global Health in 2019. https://www.who.int/news-room/spotlight/ten-threats-to-global-health-in-2019.

[149] Bekkat-Berkani R., Romano-Mazzotti L. (2018). Understanding the Unique Characteristics of Seasonal Influenza Illness to Improve Vaccine Uptake in the US. Vaccine.

[150] Kissling E., Maurel M., Pozo F., Pérez-Gimeno G., Buda S., Sève N., Domegan L., Hooiveld M., Oroszi B., Martínez-Baz I. (2025). Influenza Vaccine Effectiveness in Europe and the Birth Cohort Effect against Influenza A(H1N1)Pdm09: VEBIS Primary Care Multicentre Study, 2023/24. Eurosurveillance.

[151] Health and Social Care (HSC) Public Health Agency Respiratory Surveillance Report—Northern Ireland—Week 49: 1 December 2025–7 December 2025. https://www.publichealth.hscni.net/services-and-teams/public-health-services/health-protection/surveillance-data/respiratory-0#vaccine-effectivenes.

[152] Kirsebom F.C., Thompson C., Talts T., Kele B., Whitaker H.J., Andrews N., Abdul Aziz N., Rawlinson C., Green R.E., Quinot C. (2025). Early Influenza Virus Characterisation and Vaccine Effectiveness in England in Autumn 2025, a Period Dominated by Influenza A(H3N2) Subclade K. Eurosurveillance.

[153] Shen Y., Zhang D., Feng Z., Ma C., Shi W., Duan W., Li J., Zhang L., Wu D., Zhang J. (2026). Moderate Protection from Vaccination against Influenza A(H3N2) Subclade K in Beijing, China, September to December 2025. Eurosurveillance.

[154] Lippert J., Benjamins M., Silva A., Buscemi J. (2025). Society of Behavioral Medicine Supports Legislation to Prevent the Public Health Impact of the Flu. Transl. Behav. Med..

[155] O’Halloran A., Habeck J.W., Gilmer M., Threlkel R., Chai S.J., Hall B., Armistead I., Alden N.B., Meek J., Yousey-Hindes K. (2025). Influenza-Associated Hospitalizations During a High Severity Season—Influenza Hospitalization Surveillance Network, United States, 2024–25 Influenza Season. MMWR Morb. Mortal. Wkly. Rep..

[156] Lin J., Li C., He W. (2023). Trends in Influenza Vaccine Uptake before and during the COVID-19 Pandemic in the USA. Public Health.

[157] Ma R., Du Y., Jing W., Ma H., Wang K., Liu A., Chen S., Zhou M., Zhou Y., Su S. (2025). Seasonal Influenza Vaccination Rate and Vaccine Effectiveness among Older Adults in Mainland China: A Systematic Review and Meta-Analysis. Age Ageing.

[158] World Health Organization (2022). Guidelines for the Clinical Management of Severe Illness from Influenza Virus Infections.

[159] Hui D.S.C., Chan K.K.P. (2025). Host Immunomodulatory Interventions in Severe Influenza. J. Infect. Dis..

[160] Bonomini A., Mercorelli B., Loregian A. (2025). Antiviral Strategies against Influenza Virus: An Update on Approved and Innovative Therapeutic Approaches. Cell. Mol. Life Sci..

[161] Rosero C.I., Gravenstein S., Saade E.A. (2025). Influenza and Aging: Clinical Manifestations, Complications, and Treatment Approaches in Older Adults. Drugs Aging.

[162] Hamad A., Abdelhai M.Y., Elsherbiny M., Abdelwahed A., Tolba H., Chembolu S., Yasin F., Ibrahim R., Joseph S., Almuhanadi T. (2024). Qatar’s Assisted Home Hemodialysis Program: A Beacon of Hope for the Vulnerable Patient. Qatar Med. J..

[163] Ansari A., Taffaro A., McSween Z., Lu J., Huang A., Lazarescu R. (2025). Battling Influenza in Elderly Patients: A Case of Severe Influenza A with Complications in a High-Risk Patient. Cureus.

[164] Xu Y., Qi L., Yang J., Duan Y., Jiang M., Sun Y., Cao Y., Wu Z., Tang W., Feng L. (2025). Factors Affecting the Severity of Respiratory Infections: A Hospital-Based Cross-Sectional Study. BMC Infect. Dis..

[165] Tu J., Yin Z., Guo J., He F., Long F., Yin Z. (2020). Acitretin Inhibits IL-17A-Induced IL-36 Expression in Keratinocytes by down-Regulating IκBζ. Int. Immunopharmacol..

[166] Mizuno K., Morizane S., Takiguchi T., Iwatsuki K. (2015). Dexamethasone but Not Tacrolimus Suppresses TNF-α-Induced Thymic Stromal Lymphopoietin Expression in Lesional Keratinocytes of Atopic Dermatitis Model. J. Dermatol. Sci..

[167] Gong W., Guo X., Zhang Y. (2018). Depletion of MicroRNA-373 Represses the Replication of Hepatitis C Virus via Activation of Type 1 Interferon Response by Targeting IRF5. Yonsei Med. J..

[168] Codo A.C., Davanzo G.G., Monteiro L.d.B., de Souza G.F., Muraro S.P., Virgilio-da-Silva J.V., Prodonoff J.S., Carregari V.C., de Biagi Junior C.A.O., Crunfli F. (2020). Elevated Glucose Levels Favor SARS-CoV-2 Infection and Monocyte Response through a HIF-1α/Glycolysis-Dependent Axis. Cell Metab..

[169] Tian M., Liu W., Li X., Zhao P., Shereen M.A., Zhu C., Huang S., Liu S., Yu X., Yue M. (2021). HIF-1α Promotes SARS-CoV-2 Infection and Aggravates Inflammatory Responses to COVID-19. Signal Transduct. Target. Ther..

[170] Winheim E., Rinke L., Lutz K., Reischer A., Leutbecher A., Wolfram L., Rausch L., Kranich J., Wratil P.R., Huber J.E. (2021). Impaired Function and Delayed Regeneration of Dendritic Cells in COVID-19. PLoS Pathog..

[171] Beserra D.R., Alberca R.W., Branco A.C.C.C., de Mendonça Oliveira L., de Souza Andrade M.M., Gozzi-Silva S.C., Teixeira F.M.E., Yendo T.M., da Silva Duarte A.J., Sato M.N. (2022). Upregulation of PD-1 Expression and High SPD-L1 Levels Associated with COVID-19 Severity. J. Immunol. Res..

[172] Bonam S.R., Hu H., Bayry J. (2022). Role of The PD-1 and PD-L1 Axis in COVID-19. Future Microbiol..

[173] Yin X., Riva L., Pu Y., Martin-Sancho L., Kanamune J., Yamamoto Y., Sakai K., Gotoh S., Miorin L., De Jesus P.D. (2021). MDA5 Governs the Innate Immune Response to SARS-CoV-2 in Lung Epithelial Cells. Cell Rep..

[174] Stoy N. (2021). Involvement of Interleukin-1 Receptor-Associated Kinase 4 and Interferon Regulatory Factor 5 in the Immunopathogenesis of SARS-CoV-2 Infection: Implications for the Treatment of COVID-19. Front. Immunol..

[175] Gubernatorova E.O., Gorshkova E.A., Polinova A.I., Drutskaya M.S. (2020). IL-6: Relevance for Immunopathology of SARS-CoV-2. Cytokine Growth Factor Rev..

[176] Del Valle D.M., Kim-Schulze S., Huang H.-H., Beckmann N.D., Nirenberg S., Wang B., Lavin Y., Swartz T.H., Madduri D., Stock A. (2020). An Inflammatory Cytokine Signature Predicts COVID-19 Severity and Survival. Nat. Med..

[177] Majidpoor J., Mortezaee K. (2022). Interleukin-6 in SARS-CoV-2 Induced Disease: Interactions and Therapeutic Applications. Biomed. Pharmacother..

[178] Sarkar S., Ratho R.K., Singh M., Singh M.P., Singh A., Sharma M. (2023). Role of Viral Load and Host Cytokines in Determining the Disease Severity of Respiratory Syncytial Virus-Associated Acute Lower Respiratory Tract Infections in Children. Jpn. J. Infect. Dis..

[179] Wiseman D.J., Thwaites R.S., Drysdale S.B., Janet S., Donaldson G.C., Wedzicha J.A., Openshaw P.J., Nair H., Campbell H., Shi T. (2020). Immunological and Inflammatory Biomarkers of Susceptibility and Severity in Adult Respiratory Syncytial Virus Infections. J. Infect. Dis..

[180] Iftimie S., Gabaldó-Barrios X., Penadés-Nadal J., Canela-Capdevila M., Piñana R., Jiménez-Franco A., López-Azcona A.F., Castañé H., Cárcel M., Camps J. (2024). Serum Levels of Arachidonic Acid, Interleukin-6, and C-Reactive Protein as Potential Indicators of Pulmonary Viral Infections: Comparative Analysis of Influenza A, Respiratory Syncytial Virus Infection, and COVID-19. Viruses.

[181] Okabayashi T., Kariwa H., Yokota S., Iki S., Indoh T., Yokosawa N., Takashima I., Tsutsumi H., Fujii N. (2006). Cytokine Regulation in SARS Coronavirus Infection Compared to Other Respiratory Virus Infections. J. Med. Virol..

[182] Johnson H.M., Lewin A.S., Ahmed C.M. (2020). SOCS, Intrinsic Virulence Factors, and Treatment of COVID-19. Front. Immunol..

[183] Yazici O., Vanetti C., Clerici M., Biasin M. (2025). Experimental Models to Investigate Viral and Cellular Dynamics in Respiratory Viral Co-Infections. Microorganisms.

[184] Pinky L., DeAguero J.R., Remien C.H., Smith A.M. (2023). How Interactions during Viral–Viral Coinfection Can Shape Infection Kinetics. Viruses.

[185] George J.A., AlShamsi S.H., Alhammadi M.H., Alsuwaidi A.R. (2021). Exacerbation of Influenza A Virus Disease Severity by Respiratory Syncytial Virus Co-Infection in a Mouse Model. Viruses.

[186] Shinjoh M., Omoe K., Saito N., Matsuo N., Nerome K. (2000). In Vitro Growth Profiles of Respiratory Syncytial Virus in the Presence of Influenza Virus. Acta Virol..

[187] Stincarelli M.A., Arvia R., Guidotti B., Giannecchini S. (2024). Respiratory Virus-Specific and Time-Dependent Interference of Adenovirus Type 2, SARS-CoV-2 and Influenza Virus H1N1pdm09 During Viral Dual Co-Infection and Superinfection In Vitro. Viruses.

